# Addition of the Electronic Educational Material to Doctor's Face-to-Face Education Has No Additive Effects on Hypertension Control: A Randomized Single Blind Study

**DOI:** 10.1155/2020/8275945

**Published:** 2020-09-15

**Authors:** John Yang Lee, Dihua Tang, Xinhua Xiao, Xiaoping Liang, Huihon Piao, Mian Xie

**Affiliations:** ^1^Department of General Practice, Shenzhen People's Hospital, Southern University of Science and Technology (SUSTech), Shenzhen, China; ^2^Department of General Practice, Sichuan Provincial People's Hospital, University of Electronic Science and Technology of China, Chengdu, China; ^3^The Jingmi Community Health Service Center affiliated to Shenzhen Hospital of Guangzhou University of Chinese Medicine (Futian), Shenzhen, China

## Abstract

**Background:**

Patient education is effective for HTN treatment. There are many methods of patient education improving HTN control. Are there additive effects of combination of different educational methods for HTN treatment?

**Objective:**

To assess the effects of addition of the electronic educational material to doctor's face-to-face education for HTN control.

**Method:**

We designed a randomized single blind study to compare the doctor's face-to-face education alone and its combination with the electronic educational material over the cell phone. Participants were patients with a confirmed diagnosis of primary HTN. Electronic educational material over the cell phone was the intervention. Main measures were standard blood pressure measurements before and after 12 weeks of treatment.

**Result:**

The baseline characteristics of the intervention and control groups including the age, sex, SBP, DBP, and HTN control rate were not significantly different. After 12 weeks of follow-up, the blood pressure and the HTN control rate seemed worse in the combination group; however, the differences between the intervention group and the control group were not statistically significant.

**Conclusion:**

There were no additive effects in the combination of the doctor's face-to-face education and the electronic educational material over the cell phone.

## 1. Introduction

Hypertension (HTN) is the most common chronic disease in the world [1]. In addition to medication therapy, nonmedication therapies are considered valuable to combat the high blood pressure. Patient education is a common effective nonmedication therapy for HTN [2]. There are many methods of patient education effective for HTN, such as the traditional doctor's face-to-face education [3], pharmacist education [4], nurse teaching session [5], interdisciplinary education program [6], educational booklet [7], group patient education [8], telephone follow-up education [9], tailored educational program to change behaviors [10], and modern electronic methods including the educational e-mail [11], Facebook advertisement [12], message and WeChat over the cell phone [13], and educational Internet platform [14]. Can we apply many educational methods to boost the effectiveness? Are there additive effects in those educational methods to control HTN? To answer the question, it is mandatory to study the effects of combination of different methods of patient education. Because the patient education over the cell phone is popular in China, we conducted a randomized single blind study to compare the effects of the traditional doctor's face-to-face education alone as the control arm and its combination with the popular electronic educational material over the cell phone as the intervention arm.

## 2. Objective

To assess the effects of addition of the electronic educational material to doctor's face-to-face education for HTN control.

## 3. Method

The research project was conducted at the Jingmi Community Health Service Center at Futian District, Shenzhen China, from September 2019 to December 2019. Patients were included with the following criteria: (1) patients with the diagnosis certificate of primary HTN from the hospital; (2) patients currently under the care of the community health service center where the research project was conducted; and (3) patients participating in the research project voluntarily with the signed informed consent for the research. The exclusion criteria were (1) patients with secondary HTN and (2) patients with hypertensive emergency.

Using coin throwing method, 147 patients who were diagnosed with primary hypertension were randomized to two arms, the intervention arm consisting of 74 patients and the control arm consisting of 73 patients. All patients received antihypertensive drugs including calcium antagonists, angiotensin inhibitors, angiotensin receptor inhibitors, beta blockers, and diuretics, as the routine treatment. On average, 1-2 antihypertensive drugs were used for the majority of patients, and 3 antihypertensive drugs were used for the minority of patients. During the observation period, the quantity and dosage of drugs had no change, and the subjects visited the clinic 5 times averagely. Both arms received doctor's face-to-face education during the patient encounter, and the intervention arm received additional electronic educational material through WeChat and message over the cell phone biweekly. The electronic educational material was the electronic prescription of health education for HTN (see appendix A) and text message to ask patients to record home blood pressures.

The clinic systolic blood pressure (SBP) and diastolic blood pressure (DBP) were measured at the community health service center by the nurse who did not know the patient grouping (single blind) with the electronic blood pressure meter following the guideline for primary care of hypertension (2019) [15]. The upper arm blood pressure in sitting position was taken after resting for at least 5 minutes, using a standard cuff (length: 22–26 cm and width: 12 cm) except obese or the large arm patients who should use the large-size cuff. During the first visit, the blood pressures of both upper arms were measured, and the higher blood pressure reading site was taken. Blood pressure was measured at least twice with an interval of 1-2 minutes. If the difference between the SBPs or DBPs was less than or equal to 5 mmHg, the average value of the two measurements was taken; if the difference is more than 5 mmHg, the average value of the three measurements was taken. The time points to take the clinic blood pressure of the research project were at the beginning when patients entered the project and after 12 weeks of follow-up when patients ended the project.

The Excel was used to input and sort out the research data, and the SPSS 25.0 statistical software was used for statistical analysis. Because the age, systolic blood pressure, and diastolic blood pressure of the two groups were quantitative data, obeying the normal distribution, we used the mean ± standard deviation for the statistical description and the Student's *t*-test of two independent samples for difference comparison. Because the gender and HTN control status were qualitative data, we used the chi-square test (*χ*^2^) for difference analysis. *P* < 0.05 was taken as the difference was statistically significant. HTN control status was separated into two situations, the controlled (blood pressure < 140/90 mmHg) and uncontrolled (blood pressure ≥ 140/90 mmHg). The HTN control rate was calculated by the number of HTN controlled patients to divide the number of the total patients.

## 4. Result

### 4.1. The Baseline Characteristics of the Study Patients

The baseline characteristics of the intervention and control groups are summarized in Table 1. The age, sex, SBP, DBP, and HTN control rate were not significantly different between two groups (*P* > 0.05).

### 4.2. The Blood Pressure and HTN Control Rate after 12 Weeks of Follow-Up

The SBP, DBP, and HTN control rate of patients after 12 weeks of follow-up are recorded in the Table 2. Although the blood pressure value and the HTN control rate seemed worse in the combination group, the differences between the intervention group and the control group were not statistically significant (*P* > 0.05).

## 5. Discussion

Chronic diseases become the major cause of death of human being this century, and HTN is the most common chronic disease in the world [16]. Many methods have been applied to fight against HTN. Those methods could be classified as two categories, the medication treatment and nonmedication treatment. In the nonmedication treatment, patient education is considered as one of the most economic and effective methods [17].

There are many articles reporting different methods of patient education effective to treat HTN [4]. Because the combination of different medications usually resulted in additive effects to control blood pressure, doctors should think about the combination with different methods of patient education to combat HTN. However, there is rare research to deal with the effects of combination with different methods of patient education for HTN management. In 2019, a study using questionnaires demonstrated blending the face-to-face education and email education improving concordance of patients with hypertension, which, however, dealed with neither the blood pressure value nor the HTN control rate [11].

It is necessary to initiate research to identify if the additive effect exists in the combination of different methods of patient education for HTN control. Because sending the electronic educational material over the cell phone [13] is a popular, effective, and economically new method for HTN management based on recent publications, we therefore have initiated a randomized control study to compare the traditional doctor's face-to-face education as the control arm and its combination of the electronic educational material through WeChat and message over the cell phone as the intervention arm. It was planned to include a control arm without any education which was, however, denied by the ethic committee.

The results in the Table 1 showed that the baseline characteristics of the age, sex, SBP, DBP, and HTN control rate were not significantly different between the control arm and the intervention arm. It could be presumed that the intervention and control samples selected by the randomized coin throwing selection came from one population which was statistically equal for the assessment.

The control group received the traditional doctor's face-to-face education alone, and the intervention group received both the doctor's face-to-face education and the electronic educational material through WeChat and message over the cell phone. On the contrary to the original hypothesis in which we wished an additive effect in the intervention arm, after 12 weeks of follow-up, it seemed that the values of the SBP and DBP were higher, and HTN control rate was lower in the intervention arm; however, the differences between the two groups were not statistically significant.

Patient education was demonstrated as one of the most effective and economic methods to assist to control HTN [4]. Many methods of patient education were reported effective and rare negative for HTN treatment [10]. Based on our search, we did not find a similar study to ours to demonstrate effects of combination of different methods of patient education for HTN treatment. Unfortunately, our results demonstrated that the combination of the traditional doctor's face-to-face education and the electronic educational material had no additive effects for the HTN treatment. On the other hand, it seemed to be producing possible subtractive effects for blood pressure control, although the differences were not statistically significant. The negative results may present one type of combination of patient education. How about the effects of other combinations? We urge, hereby, to initiate research to compare the effects of combinations of different methods of patient education for the treatment of HTN and other chronic diseases in the world.

## 6. Strength and Limitations

The strength of the study include the randomized and single-blinded methodologies that simply decrease the confounding factors to compare the intervention arm and control arm. The limitations of the study include the limited sample size and no presentation of home blood pressures. We would like to extend the research in a large hypertensive population in the future. Although we asked patients to record the home blood pressures, the quality and quantity of the data for home blood pressures were not good enough for analysis to be included in the paper.

## 7. Conclusion

There were no additive effects in the combination of the doctor's face-to-face education and the electronic educational material over the cell phone.

## Figures and Tables

**Table 1:** The baseline characteristics of intervention and control groups.

Characteristics	Intervention (*N* = 74)	Control (*N* = 73)	*P* value
Age: mean years (SD)	66.26 ± 13.05	64.52 ± 11.56	0.395
Sex: number (%)			0.366
Male	44 (59.50)	38 (52.10)	
Female	30 (40.50)	35 (47.90)	
SBP: mean mmHg (SD)	133.91 ± 14.00	130.81 ± 13.66	0.177
DBP: mean mmHg (SD)	78.12 ± 8.78	76.03 ± 12.12	0.232
HTN control: number (%)	57 (76.00)	59 (80.80)	0.573

**Table 2:** The blood pressure and HTN control rate of intervention and control groups after 12 weeks of follow-up.

Characteristics	Intervention (*N* = 74)	Control (*N* = 73)	*P* value
SBP: mean mmHg (SD)	135.39 ± 16.76	130.92 ± 13.58	0.078
DBP: mean mmHg (SD)	79.19 ± 11.12	77.85 ± 9.48	0.433
HTN control: number (%)	46 (62.20)	56 (76.70)	0.056

## Data Availability

The data used to support the findings of this study are available from the corresponding author upon request.

## Appendix

### A. Electronic Prescription of Health Education for Hypertension (the Original Text Was in Chinese)

#### A.1. Definition

Hypertension is a syndrome that elevated blood pressure is the main clinical manifestation with or without multiple cardiovascular risk factors.

#### A.2. Health Guidance

(1) Diet:
  1. Sugar: sugar supplies energy for human being. It is recommended to eat complex sugar, such as starch, corn, whole wheat, and eat less monosaccharides such as glucose, fructose, and sucrose.
  2. Fat: limit fat intake and recommend using plant oil when cooking.
  3. Protein: proper intake of protein is recommended, for instance, fish protein 2-3 times a week, but if you are complicated with renal insufficiency, protein intake should be limited.
  4. Mineral: eat more potassium- and calcium-rich food but low sodium food, such as potatoes, eggplant, kelp, lettuce, milk, yogurt, and shrimp skin.
  5. Salt: limit salt intake. Strictly control the salt content in food, and daily salt intake should be gradually reduced to less than 5 g daily.
  6. Vitamin: eat more fresh vegetables and fruits. Eat no less than 400 g of fresh vegetables and 100 to 200 g of fruits every day.
  7. Others: appropriate increase in seafood intake, such as kelp, laver, and sea fish.
(2) Exercise: the aerobic exercise with the low to moderate intensity is recommended for hypertensive patients. Therefore, we recommend walking and Tanjiquan.
  1. Walking: 80∼120 steps min is suitable. If you feel energetic, you can jog. It is better to walk and jog alternately.
  2. Taijiquan: you can choose some of the most natural and relaxing movements in Taijiquan and pay attention to avoid the more difficult movements such as the independent lower limb standing and left and right leg kicking. When practicing, try to relax, breathe smoothly, and have no distractions. Generally speaking, for patients with hypertension during exercise, heart rate + age = 170 is appropriate.
(3) Mental Care.
    You can change your behavior to cultivate a good ability to adapt to the natural environment and society, avoiding emotional excitement, excessive tension and anxiety, and being calm when in trouble. When there is greater mental pressure, you should try to release it, pour it out to friends and relatives. You are encouraged to participate in relaxed and happy spare time activities. You should keep your mental health with music, flowers, and so on, so that you can live in the best state and maintain a stable blood pressure.
(4) Medication Precautions.
    According to the doctor's advice, take the medicine with the right amount and interval time, and do not stop or change the dosage at will.
(5) Regular examination and follow-up visit.
    Return visit once a month. If you have dizziness, headache, etc., please seek the medical care at any time.
